# Time-lapse analysis of potential cellular responsiveness to Johrei, a Japanese healing technique

**DOI:** 10.1186/1472-6882-5-2

**Published:** 2005-01-24

**Authors:** Ryan Taft, Dan Moore, Garret Yount

**Affiliations:** 1 Research Institute, California Pacific Medical Center, San Francisco, USA; 2 Department of Biostatistics and Epidemiology, University of California San Francisco, San Francisco, USA

## Abstract

**Background:**

Johrei is an alternative healing practice which involves the channeling of a purported universal healing energy to influence the health of another person. Despite little evidence to support the efficacy of such practices the use of such treatments is on the rise.

**Methods:**

We assessed cultured human cancer cells for potential responsiveness to Johrei treatment from a short distance. Johrei treatment was delivered by practitioners who participated in teams of two, alternating every half hour for a total of four hours of treatment. The practitioners followed a defined set of mental procedures to minimize variability in mental states between experiments. An environmental chamber maintained optimal growth conditions for cells throughout the experiments. Computerized time-lapse microscopy allowed documentation of cancer cell proliferation and cell death before, during and after Johrei treatments.

**Results:**

Comparing eight control experiments with eight Johrei intervention experiments, we found no evidence of a reproducible cellular response to Johrei treatment.

**Conclusion:**

Cell death and proliferation rates of cultured human cancer cells do not appear responsive to Johrei treatment from a short distance.

## Background

Relatively little documentation supports a scientific basis for alternative healing therapies involving the manipulation of a purported healing energy associated with the body. Despite this paucity of evidence, such *energy healing *modalities are becoming increasingly popular. Recent surveys indicate the majority of the United States public has used alternative medical therapies, and that energy healing therapies are among the fastest growing complementary and alternative medicine treatments [1,2]. Johrei is one such energy healing practice with origins in Japan. Johrei was founded by Mokichi Okada in the 1920's and is now practiced worldwide with significant numbers of adherents in Asia, Europe and the Americas. Practitioners of Johrei believe it possible to improve the health of others by directing a universal healing energy toward them. The Johrei philosophy, as it relates to healing energy, emphasizes what can be described as "spiritual purification." Other aspects of the Johrei philosophy include fostering an appreciation of beauty, and a form of organic farming.

Two scientific studies evaluating Johrei healing practices have found provocative results. In one, Johrei was shown to have a beneficial affect on the mood of practitioners [3]. Results from a second study suggested that those practicing Johrei may display an immune profile consistent with stress reduction [4]. Clinical studies evaluating potential efficacy of Johrei are ongoing [5,6]. An in vitro study revealed an apparent influence of Johrei treatment on the germination rate of irradiated seeds but the study did not follow standard scientific methods [7]. To investigate whether Johrei can have a direct effect on human cells, we exposed cultured cancer cells to Johrei treatment from a short distance. Johrei treatments were maintained continuously for four hours by teams of Johrei practitioners. Experiments were conducted using strict blinding procedures, exceeding standards commonly used to evaluate conventional therapeutics.

## Methods

### Overall study design

For each session Johrei practitioners were asked to direct healing intention toward cell cultures in a temperature, CO_2_, and humidity controlled time-lapse microscope incubator chamber. Johrei practitioners participated in teams of two, alternating every half hour such that a total of 4 hours of Johrei treatment was delivered. A total of eight control and eight Johrei intervention experiments were accomplished. Johrei treatments were initiated after 4 hours of baseline data had been collected and the observation period extended for a total of 22 hours. We quantified tumor cell death and proliferation throughout the observation period. We also quantified the rate of cellular emigration (i.e. how many tracked cells left the microscopic field per hour) in order to accurately assess cell population dynamics (i.e. cell death and proliferation rates).

### Blinding procedures

Experiments were conducted with blinding applied to each of the scientists based on previously reported methods [8]. The experimental protocol was divided among scientists such that those responsible for preparation of the cell cultures, data acquisition, and data analysis were blind to each other's activities and results until data analysis was complete.

### Cell culture

As the target of Johrei healing intentionality in these studies we used a human cell line derived from a brain tumor biopsy specimen from a patient with glioblastoma multiforme (SF188 GBM). This cell culture model is used widely and can show responsiveness to conventional therapeutic agents including ionizing radiation and chemotherapeutics. SF188 GBM cells were grown in RPMI media supplemented with 10% fetal bovine serum, 100 U/ml penicillin, 100 μg/ml streptomycin sulfate, and 0.25 μg/ml amphotericin. A fresh aliquot of cryogenically preserved cells was thawed at the start of each experiment to ensure uniformity in the genetic profile of the target cells throughout the experiments. Cells were plated at a density of 5,000 cells per well in six-well culture plates and allowed to grow uninterrupted for 20 hours in a humidified incubator maintained at 37°C and 5% CO_2 _before beginning each time-lapse experiment.

### Time-lapse microscopy

For each experiment cell cultures were transferred from the incubator to a time-lapse microscope in our laboratory equipped with a heated stage, CO_2 _chamber, and a plexi-glass environmental chamber (Axiovert 200; Zeiss, Gottingen, Germany). Cell cultures were maintained at routine incubation settings (37°C, 5% CO_2_) and optimum humidity. Temperature and CO_2 _concentration were independently maintained using digital controlling units (Zeiss, Gottingen, Germany). Two sets of phase contrast images (100 X magnification) from each well were taken in 300 second intervals using a Cohu 2600 Series compact monochrome interline transfer CCD camera. Images were acquired during a four-hour baseline period and then continuously for another 18 hours. An Openlab software automation (Improvision, Lexington, MA) drove the camera and stage movements, and compiled the acquired phase images. Images were subsequently processed as Quicktime movies using Openlab.

### Johrei intervention

Johrei practitioners were selected based on experience and willingness to participate by the Center for the Science of Life, a Johrei organizational body in the United States. Five Johrei practitioners with a minimum of 17 years experience participated in the experiments in teams of two. Johrei treatment was administered by the practitioner teams for a total of four hours, beginning after the four-hour baseline period. This exceptionally long treatment period (4 hours) was chosen as the highest "dose" that was practical within the experimental model. Each practitioner treated the cells for a total of 2 hours per experiment, switching every half hour with the team member. Treatment began with one of the two practitioners being seated in front of the time-lapse microscope and raising one hand toward the cellular target. One hand remained raised toward the cell cultures for the duration of any given Johrei treatment. Johrei treatments were delivered from a distance of 6 – 9 cm, from outside the plexiglass environmental chamber.

In collaboration with The Center for the Science of Life, we developed a set of standard mental procedures for practitioners to follow to minimize variability in the mental states among practitioners. These mental cues were used by all practitioners in all experiment. The mental cues can be summarized briefly as: 1) establishing a connection to the divine, 2) consciously relaxing body and mind, 3) visualizing healing energy penetrating the cellular target, 4) taking enjoyment in participating in the experiment, 5) maintaining a feeling of gratitude.

### Time-lapse microscopy data analysis

Every cell in the initial microscopic field was identified and numbered. For each experiment, we counted the initial number of cells in each of 12 microscopic fields. All numbered cells and their progeny were then tracked for the duration of their onscreen viability using compiled Quicktime movies. Each cell's life events were identified using a modified version of a previously described cell pedigree system [9]. Cells identified as dead at the start of the video or that entered the microscopic field after the initial frame were not included in this analysis. Cataloged data was entered into a Microsoft Excel spreadsheet (using blinding codes) for further analysis. Cell deaths and divisions occurring per half hour were recorded.

### Statistical analysis

Statistical analysis was based on a model which categorizes a cell as engaged in any one of four activities at any time: 1) division, resulting in an additional cell (division), 2) death, resulting in the loss of a cell (death), 3) movement out of the microscopic field (emigration), or 4) the cell can remain unchanged [10]. Under this model three transition probabilities plus the total number of cells at a previous time determine the number of cells at a future time. The expected number of cells at time t, N(t), is given by the equation

N(t) = N(t-1) exp(λ(t) - μ(t) - ν(t))],

where λ(t), μ(t) and ν(t) are the transition probabilities for division, death and emigration, respectively, at time t. We estimated the transition probabilities in one-hour time blocks. For example, the estimate for λ(t) is

λ(t) = [ln(N(t) + div(t)) - ln(N(t-1))],

where div(t) is the number of divisions during (t-1, t). A similar equation was used for estimating death and emigration transition probabilities at each hour.

Statistical analyses focused on two types of comparisons: comparisons within the Johrei treatment experiments, where division and death rates (transition probabilities) pre-treatment were compared with those during and post-treatment; and comparisons between Johrei and control experiments at similar times. Data from each experiment was pooled at 30 minute time intervals. Each of the cellular events (division, death or emigration) occurs infrequently to each cell, so it is necessary to pool results from many cells in order to have nonzero data. Additionally, in instances where few events were recorded due to relatively few cells being observed it was necessary to pool over several time intervals in order to have enough data to perform statistical tests.

## Results

Overall we documented the behavior of 336 cells in the eight control experiments and 351 cells in the eight Johrei intervention experiments. Initial numbers of cells per microscopic field ranged from 5 to 10; the average cell count per field was 7.0 for controls and 7.3 for Johrei. The numbers of cells per experiment ranged from 40 to 48 with averages of 42.0 and 43.9 for controls and Johrei. Observation over 22 hours showed 312 divisions, 15 deaths and 17 emigrations in the control experiments and 316 divisions, 21 deaths and 7 emigrations in the Johrei experiments. Differences in number of divisions or deaths is not significant (p = 0.19 for divisions and p = 0.37 for deaths based on chi-square test comparing frequencies). While the difference in emigrations is statistically significant (p = 0.03, chi-square test comparing frequencies), this was largely affected by a difference present in the 0–4 hour baseline period making it unlikely that the observed effect is due to Johrei treatment.

Experimental treatments (control or Johrei) were confined to a four-hour period starting after collection of four hours of baseline data. During the four-hour treatment period the numbers of divisions were 84 (control), 59 (Johrei); deaths were 4 (control), 5 (Johrei); and emigrations were 0 (control) and 2 (Johrei). Taking into account the numbers of cells at the start of the treatment period, only the difference in divisions is statistically significant (p = 0.018 based on comparing the division rates during the treatment period). This result is based on pooling observations from all cells over the four-hour period and the p-value is based on an assumption that counts observed follow a Poisson distribution. It is possible that counts do not follow a Poisson distribution and that their variability is greater than that expected for Poisson counts. To examine this we calculated division rates separately for each of the eight control experiments and eight Johrei experiments and then compared these rates using the nonparametric Mann-Whitney rank sum test. This analysis confirmed the result based on the total counts (p = 0.016 for Mann-Whitney test).

**Figure 1:**
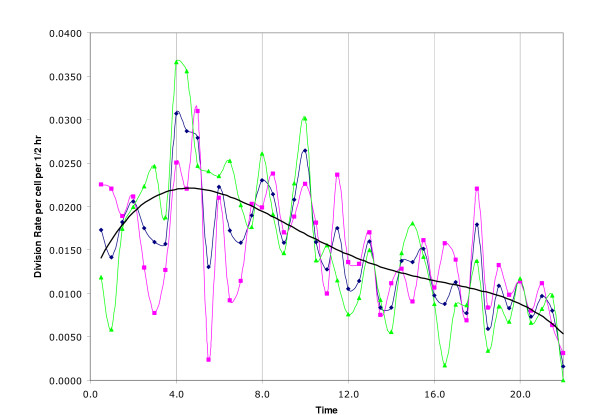
Rates of cell division are shown as plotted against time in hours. The plot depicts a decrease in divisions in Johrei treated cells (pink line) during the 4.0–8.0 treatment period which is statistically offset by a similar dip in the control period. Control and pooled data are depicted by green and blue lines respectively. The solid black line depicts the fit of a 4^th ^degree polynomial to half-hourly cell division rates for all experiments.

**Figure 2:**
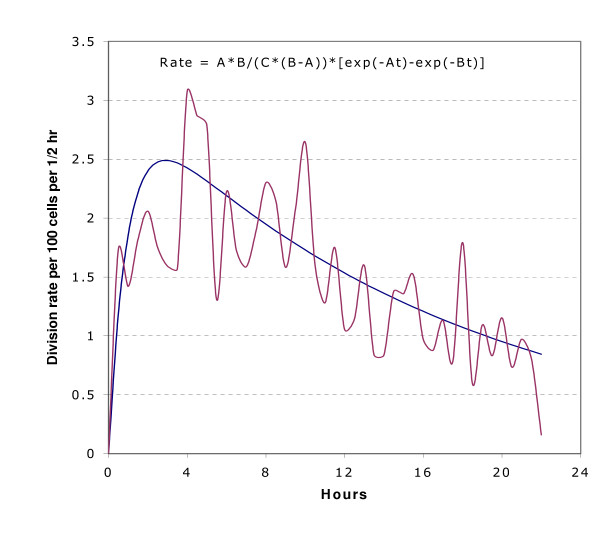
The fit of the sum of two exponentials to pooled division rate data for all experiments is depicted. The maroon line is pooled data and the blue line is fit.

We also tried fitting parametric models to the data in an effort to gain degrees of freedom for performing statistical tests. For example, we tried fitting low order polynomials to division rates over time, but the fits were unsatisfactory, as judged by assuming counts were Poisson distributed with rates predicted from the polynomial fit. The fits were particularly deficient in following the rapid initial rise in division rates over the first four hours of baseline data (Figure 1). We obtained similar results attempting to fit a sum of exponentials model (Figure 2).

**Figure 3:**
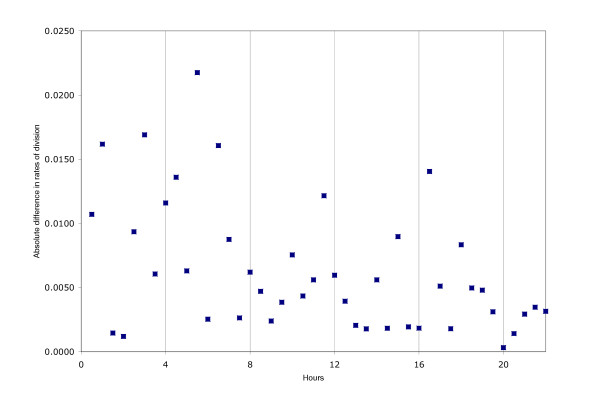
Absolute differences in half-hourly rates of cell division for all experiments are depicted. The plot suggests that differences between control and Johrei experiments were largest during the first 8 hours and that the differences during Johrei treatment (4–8 hrs) were not statistically larger than those during the previous baseline time period, when the treatment conditions were identical.

The division rates for control and Johrei experiments plotted in Figure 1 suggest another method of comparison. The experimental conditions should be identical during the 0–4 hour baseline period, yet we see wide variation in division rates for the two conditions. We can measure the variance between control and Johrei experiments at each half-hour time point and use the pooled value, over the first eight time points, as a baseline standard. We computed the pooled variances for the 4–8 hour time period (treatment) and compared the two using an F-test for equality of variances. The pooled variance for the 0–4 hours period was 5.8 × 10^-5 ^compared to 6.8 × 10^-5 ^for the 4–8 hours period. The ratio of these is 1.17 which is not statistically significant (p = 0.41 based on F distribution with numerator and denominator df = 8). This suggests that the discrepancies between the rates during the 4–8 hour period are statistically no larger than those observed during the 0–4 hour period when there were no treatment differences. This comparison requires no assumption of Poisson variability of counts and takes advantage of a priori knowledge that treatment conditions were identical in the first four hours of the experiments. The only assumption is that the variances arise from normally distributed data, so this requirement was tested. A Mann-Whitney comparison of the ranks of the absolute differences in control and Johrei rate during 0–4 hours vs. 4–8 hours give a p-value of 0.92. Thus, there appears to be no evidence that differences in rates of division during 4–8 hours for control and Johrei were any different from those during 0–4 ours. A plot of these absolute differences is shown in Figure 3. The original data are provided as supplementary material [see Additional file 1].

## Discussion

Our initial examination of the data suggested that Johrei may have affected the rate of division of cultured human brain tumor cells. This analysis, however, was based on the assumption that the baseline period in both the control and Johrei treated cultures were statistically identical with respect to cell divisions and cell deaths. Subsequent analysis revealed that a significant difference existed in the baseline period between control and Johrei treated samples, with Johrei cultures exhibiting fewer divisions. Taking this into account it appears that there was no observable effect of Johrei on these cell behaviors.

The failure to observe evidence of a reproducible cellular response to Johrei treatment is consistent with prior studies in our laboratory evaluating another popular energy medicine modality, external Qigong. Like Johrei, practitioners of external Qigong generally claim the ability to emit or direct healing energy to treat patients. Qigong practices originate from China and are based on the manipulation of a purported healing energy called "Qi." Our prior study investigated the ability of experienced Qigong practitioners to enhance the growth of normal human brain cells in culture as measured by a colony-forming efficiency assay. Following a rigorously designed protocol with randomization, blinding and controls for variability, we did not observe reproducible effects of external Qigong treatment on the growth of these cells [11]. Such "negative" data do not negate the possible therapeutic effects of such practices. Cultured cells offer an incomplete system that may not be sensitive to treatments of this kind. More complete models (e.g., a clinical research model) may be more useful to evaluate Johrei treatment for a variety of reasons.

## Conclusion

Cell death and proliferation rates of cultured human cancer cells do not appear responsive to Johrei treatment from a short distance.

## Competing interests

The Center for the Science of Life, a Johrei organizational body in the United States, has provided funding for this work.

## Authors' contributions

RT and GY conceived of the study and participated in its design, coordination and implementation. DM performed statistical analysis. All authors read and approved the final manuscript.

## Pre-publication history

The pre-publication history for this paper can be accessed here:



## Supplementary Material

Additional File 1 Johrei Experimental Data. The supplementary file includes the raw data for all experiments, including number of events for cell death, division, and emigration by half-hourly intervals and cumulative totals. Click here for file
